# Supplementary material for: Nomograms Combining PHI and PI-RADS in Detecting Prostate Cancer: A Multicenter Prospective Study
Source: J Clin Med. 2023 Jan 1;12(1):339. doi: 10.3390/jcm12010339 (PMC9821430; doi:10.3390/jcm12010339)
Supplement: Supplementary file 1 [file jcm-12-00339-s001.zip › jcm-2116186-supplementary.pdf]

Table S1 Demographic characteristics of different hospitals

Characteristics	Hospital 1	Hospital 2	Hospital 3	*P* value
N	29	222	42	/
Age (years), median (IQR)	69.00(64.50-74.00)	66.00(59.00-72.00)	67.50(60.00-74.00)	0.121
TPSA (ng/ml), median (IQR)	11.59(8.81-16.51)	8.09(5.84-11.46)	8.52(5.85-12.11)	0.003
fPSA (ng/ml), median (IQR)	1.13(0.93-2.38)	1.14(0.79-1.59)	1.12(0.68-1.88)	0.390
P2PSA (ng/ml), median (IQR)	17.89(14.14-25.68)	19.77(13.33-30.38)	12.21(8.01-17.49)	0.000
PHI, median (IQR)	43.72(32.23-63.70)	48.92(38.32-72.70)	34.39(25.22-49.74)	0.000
f/T, median (IQR)	0.12(0.09-0.20)	0.14(0.10-0.19)	0.13(0.09-0.19)	0.518
%P2PSA, median (IQR)	1.61(0.89-2.02)	1.83(1.36-2.50)	1.29(0.91-1.71)	0.000
PV (ml), median (IQR)	58.21(32.36-78.77)	40.64(38.32-72.70)	50.10(25.22-49.74)	0.026
PI-RADS, n (%)				0.188
≤2	10(34.5)	84(37.8)	23(54.8)	
3	8(27.6)	72(32.4)	12(28.6)	
≥4	11(37.9)	66(29.7)	7(16.7)	
PHID, median (IQR)	0.72(0.46-1.63)	1.22(0.68-2.31)	0.74(0.35-1.25)	0.000
PSAD (ng/ml^2), median (IQR)	0.20(0.15-0.31)	0.19(0.12-0.32)	0.16(0.11-0.27)	0.174

IQR: interquartile range; TPSA: total prostate-specific antigen; fPSA: free prostate-specific antigen; P2PSA: [-2]pro-prostate-specific antigen; PHI: prostate health index; f/T: free/total prostate-specific antigen; %P2PSA: defined as [(P2PSA/fPSA) × 100]; PV: prostate volume; PI-RADS: Prostate Imaging-Reporting and Data System; PSAD: prostate-specific antigen density; P value for the comparison between 3 hospitals.

Figure S1

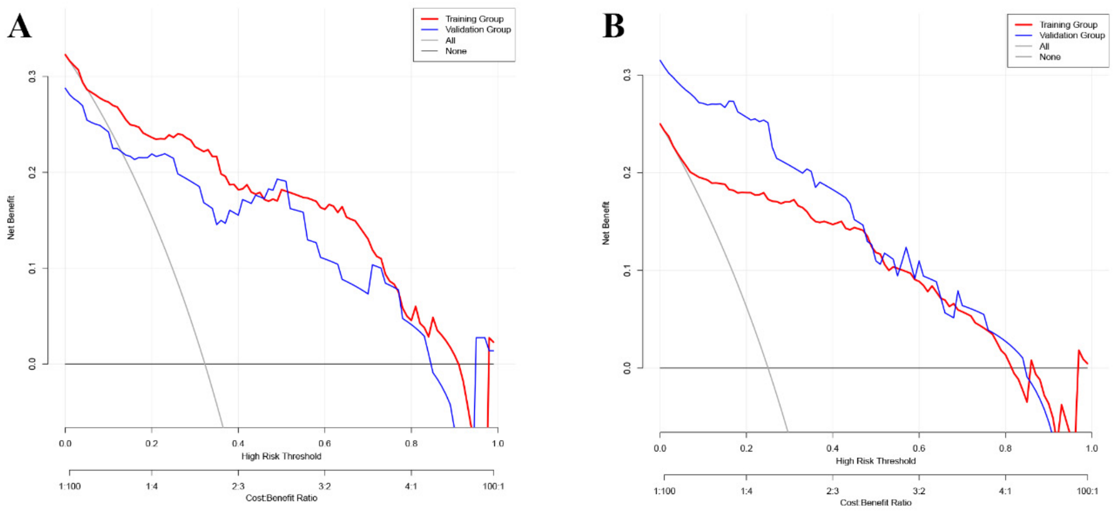

Figure S1: Decision curve analysis for the PCa nomogram in the training and validation cohorts (A) and for CSPCa nomogram in the training and validation cohorts (B). The y-axis measures the net beneft, the black line represents the assumption of PCa-none-patients (A) and CSPCa-none-patients (B), the gray line represents the assumption of PCa-all-patients (A) and CSPCa-none-patients (B), the red line represents the training cohort, and the blue line represents the validation cohort. PCa: prostate cancer; CSPCa: clinically significant prostate cancer, defined as Gleason Grade≥2.
